# Design of a AFLP-PCR and PCR-RFLP test that identify the majority of discrete typing units of *Trypanosoma cruzi*

**DOI:** 10.1371/journal.pone.0237180

**Published:** 2020-08-04

**Authors:** Lynneth Rivas-García, Manuel Alejandro Carballo-Amador, Carlos Alberto Flores-López

**Affiliations:** Facultad de Ciencias, Universidad Autónoma de Baja California, Ensenada, Baja California, Mexico; University of Helsinki, FINLAND

## Abstract

**Background:**

Chagas disease, caused by the intracellular parasite *Trypanosoma cruzi*, is one of the most important parasitological infections in the Americas. It is estimated to infect approximately 6 million people from mostly low income countries in Latin America, although recent infections have been reported in southern US states. Several studies have described an extensive genetic diversity among *T*. *cruzi* isolates throughout its geographic distribution in the American continent. This diversity has been correlated with the pathology developed during an infection. However, due to a lack of a single reliable test, current diagnosis practices of the disease are not straightforward since several different tests are applied. The use of current genomic sequence data allows for the selection of molecular markers (MM) that have the ability to identify the Discrete Typing Unit (DTU) of *T*. *cruzi* in a given infection, without the need of any sequencing reaction.

**Methodology/principal findings:**

Applying three criteria on the genomic sequencing data of four different phylogenetic lineages of *T*. *cruzi*, we designed several molecular tests that can be used for the molecular typing of the parasite. The criteria used were: (1) single-copy orthologs of *T*. *cruzi*, (2) *T*. *cruzi* unique loci, and (3) *T*. *cruzi* polymorphic loci. All criteria combined allowed for the selection of 15 MM, 12 of which were confirmed to be functional and replicable in the laboratory with sylvatic samples. Furthermore, one MM produced distinct polymerase chain reaction (PCR) amplicon sizes among distinct *T*. *cruzi* DTUs, allowing the use of a AFLP-PCR test to distinguish DTUs I, II/IV, V and VI. Whereas two MM can differentiate DTUs I, II, IV and V/VI out of the six current DTUs with a PCR-RFLP test.

**Conclusions/significance:**

The designed molecular tests provide a practical and inexpensive molecular typing test for the majority of DTUs of *T*. *cruzi*, excluding the need to perform any sequencing reaction. This provides the scientific community with an additional specific, quick and inexpensive test that can enhance the understanding of the correlation between the DTU of *T*. *cruzi* and the pathology developed during the infection.

## Introduction

*Trypanosoma cruzi* is the etiological agent of American trypanosomiasis, also known as Chagas disease [1]. No vaccine has been developed for this infection, and the two drugs available to treat it are of limited use and may present severe secondary effects [2]. The pathology associated with the infection consists of two stages: an acute phase (the first 2–3 weeks of infection) characterized by high parasitemia and a chronic phase (10–30 years after infection) defined by low parasitemia and syndromes mostly associated with heart failure, megacolon, and megaesophagus [1]. The disease is mostly considered to be endemic to Latin America, where the parasite is usually transmitted by its vector-mediated transmission or via the oral path of transmission [1]. However, recent studies have found autochthonous cases of infection in the southeastern United States [3, 4]. The disease is currently estimated to affect approximately 6 million people [5]. However, this number is challenging to assess, given the limited amount of data, inefficient public health systems.

Currently, the World Health Organization (WHO) considers direct parasitological tests of blood smears for the acute phase and any two different serologic tests (enzyme-linked immunosorbent assay [ELISA], indirect immunofluorescence, and/or indirect hemagglutination) for the chronic phase to be the standard diagnostic practices for the infection, as a result of the lack of a gold-standard test [5, 6]. However, several alternative diagnostic procedures have been developed (xenodiagnosis, blood smears, strout, microstrout, microhematocrit, hemoculture, PCR, and quantitative PCR [qPCR]) [7], since the standard diagnosis continues to be inaccurate. Some of these are considered “indirect” tests because they do not directly detect the presence of the parasite, but rather detect the presence of antibodies, which cannot discriminate among cases where the host immune system might have neutralized the pathogen. The development of “direct” diagnostic tests, like a PCR targeted at amplifying *T*. *cruzi* unique loci, has been prioritized [8, 9].

In addition to the nature of the test, the genetic background of the parasite needs to be considered. The genetic diversity of this mostly asexual parasite is classified into what is known as discrete typing units (DTUs), labeled I to VI, with a recent seventh genetic lineage termed TcBat [10–12]. However, stringent phylogenetic analyses can reduce this classification system into three or four monophyletic clades, depending on the MM used (three with mitochondrial loci and four with nuclear loci) (S1 Table) [13, 14]. To date, three of the four monophyletic clades are represented in the annotated genomes sequenced [15–17]. There can be as much as a 20–40% difference in genome content among some phylogenetic groups [17], which highlights the potential biological differences among the different *T*. *cruzi* DTUs presently circulating in nature.

The characterization of this parasite’s diversity is essential, since a correlation of the pathology of the infection with the genetic background of the parasite has been reported [18–23]. Although other factors appear to play a role in the severity of a given infection [24], identifying the given genetic background of the parasite in a given human infection is crucial for further understanding of the disease.

The potential medical benefit attained from sequencing genomes of human pathogens cannot be undervalued. For *T*. *cruzi*, the first genomic sequence became available almost 15 years ago [15], marking an enormous accomplishment for neglected tropical diseases. A project that was specially challenging to accomplish due to the vast diversity of surface protein coding genes intrinsic to *T*. *cruzi* genomes. Fortunately, currently there are several annotated genomes of *T*. *cruzi*, representing a significant percentage of the total genomic diversity described so far [25]. It is vitally significant to identify molecular markers with the ability to distinguish the genetic background of a pathogen of interest in the absence of DNA sequencing.

To accomplish this objective, three criteria were targeted for the design of the molecular tests: single-copy orthologs, *T*. *cruzi* species-specific loci, and genetically diverse loci. The rationales behind each of these criteria were as follows: (1) selecting single-copy orthologs would prevent the amplification of paralogs, since the use of paralogs has the potential of amplifying multiple amplicons due to mispriming during the PCR; (2) selecting loci unique to *T*. *cruzi* would ensure a species-specific molecular typing test for the infection by *T*. *cruzi*; and finally, (3) selecting genetically diverse loci would allow the identification of the DTUs of *T*. *cruzi* by either distinctive PCR amplicon sizes or the development of unique restriction fragment length digestion profiles. This would potentially produce MM with the power to differentiate the major DTUs of *T*. *cruzi* without a need to sequence the amplicons, once all three criteria are combined. Resulting in an alternative molecular typing test that is quicker and independent to a Sanger sequencing based reaction, which although is comparable in cost, is not necessarily a readily available option in most Latin American laboratories.

## Materials and methods

### Design of molecular tests

To select the loci with the traits required for the molecular typing test to be species specific (i.e., to amplify solely in *T*. *cruzi*) and accurate (i.e., to be able to differentiate the majority of DTUs of the parasite), genome sequences of the four annotated *T*. *cruzi* strains available in February 2017 at TriTrypDB were used [25]. The strains analyzed were Sylvio classified as DTU I [17], Dm28c cataloged as DTU I [26], Esmeraldo classified as DTU II [15], and CL Brener classified as DTU VI [15].

Selection of single-copy orthologs was accomplished by excluding paralogs from the selection. Any self-BLAST hits with an E value <0.05 were excluded [27]. Selection of loci unique to *T*. *cruzi* was attained by confirming the uniqueness of the loci for *T*. *cruzi* in Genbank’s nr/nt database [28] and by excluding any loci that had another BLAST E value hit of <0.001 in any other taxonomic unit. To avoid mispriming to other non *T*. *cruzi* species, the number of SNPs found within the primer annealing regions were quantified by comparing these target sequences among the *Trypanosoma* genus sequence data. Finally, for the selection of genetically diverse loci, ortholog sequences from the four genomes used were aligned and orthologs were prioritized according to their diversity in terms of distinct gene sizes among DTUs and/or the highest number of SNPs among DTUs.

### Primer design and PCR

Orthologs that fulfilled all of the three criteria previously mentioned were selected as potential loci for primer design that would target their amplification via PCR. Primers were designed in Primer3 [29], where the annealing position was selected while trying to avoid polymorphic regions, hence increasing the likelihood of the primers annealing on all of the *T*. *cruzi* genetic groups. Thus, we focused on heavily conserved regions within the loci for their design, based on the sequence alignments of the four phylogenetic lineages available. In addition, the design of relatively small amplicons was favored, without sacrificing the genetic diversity recovered. This favors efficient PCR amplification from samples with poor DNA quantity, a scenario commonly encountered in chronic Chagas infections.

The PCR optimization reactions were assayed under the following PCR amplification conditions: an initial denaturation step at 95°C for 4 min, followed by 35 cycles of a 30-s denaturation step at 95°C, annealing temperature with a gradient of ±2°C, focused on the average annealing temperature for each set of primers for 50 s, extension at 72°C for 50 s, and a final extension of 5 min at 72°C. PCR was performed in a Veriti 96-Well Fast Thermal Cycler from Applied Biosystems. PCR reactions had the following volumes: 1.5 μL of 10× PCR Rxn Buffer (-MgCl_2_), 0.45 μL MgCl_2_ (50 mM), 0.3 μL dNTPs (10 mM each) (VWR Life Science Amresco), 0.75 μL for each primer (50 pmol), 0.06 μL Taq polymerase (5 U/μL) (Invitrogen), 1 μL of DNA (10 ng/μL), and DEPC water to obtain a final volume of 15 μL per sample.

The PCR amplicons were confirmed by agarose 1% (w/v) gel electrophoresis at 80 V for 30 min in a mini sub cell (BioRad) stained with SYBR Safe DNA gel stain (Invitrogen, USA). DNA fragments were visualized under ultraviolet (UV) light with the Azure c200 gel documentation platform. The PCR performance was tested using *T*. *cruzi* clone DNA samples representing all major DTUs (except for DTU III), which were kindly provided by Dr. Carlos Machado at the University of Maryland. Once the functionality of the PCR was established, DNA extracted from the digestive tracts of field collected *Dipetalogaster maxima* individuals were used. These samples were part of an independent study aimed at describing the natural infection rate of *T*. *cruzi* in this Baja California endemic species throughout a three-year study period (unpublished results). In this study the natural infection rate was determined by using two distinct *T*. *cruzi* specific MM (COII-NDI & Tc00.1047053506529.310) [13, 14]. Posteriorly, a random set of four positive *T*. *cruzi* DNA samples from this study were used to verify the efficiency of the MM.

The species specificity of the MM was tested on DNA of five distantly related taxonomical orders (Artiodactyla, Asterales, Rhabditida, Chytridiales, and Coleoptera). To further evaluate the specificity of the primers, the SNPs present within the annealing regions of all primers among other *Trypanosoma* spp. were quantified (for *T*. *rangeli*, *T*. *vivax*, *T*. *evansi*, *T*. *theileri*, and *T*. *cruzi marinkellei*).

### Discrete typing unit discrimination

Genetic loci that would produce distinct PCR amplicon sizes for each major phylogenetic group were selected as potential MM. Likewise, the polymorphic sites detected by aligning the homolog sequences from all selected *T*. *cruzi* loci were designated as potential targets to discover restriction enzymes that would selectively produce distinct length profiles via a PCR restriction fragment length polymorphism (RFLP) test. These targets were analyzed in Serial Cloner 2.6.1 to screen for potential restriction endonucleases that would differentiate among the major DTUs in each infection. The enzymatic reactions were performed according to the manufacturer’s protocol (New England BioLabs), using the amplicons of distinct *T*. *cruzi* DTUs as a template (S1 Table). The *T*. *cruzi* strains for which a DNA template was used in PCR (S1 Table) were distinct from the *T*. *cruzi* strains for which genomes were used in the primer design procedure. Hence, the PCR amplicons used for the PCR-RFLP were sequenced prior to their respective enzymatic digestion, to evaluate the conservation of restriction digestion sites among distinct strains classified within the same DTU. This was a necessary appraisal that provided only a little information into the conservation of SNPs, since very few strains of *T*. *cruzi* have had their respective genomes sequenced, severely limiting the assessment of the degree of conservation of the restriction digestion sites among the DTUs. Samples were analyzed by electrophoresis in a 3% (w/v) agarose gel at 110 V for 50 min in a mini sub cell stained with SYBR Safe DNA gel stain and were visualized with Azure c200 platform.

For the amplified fragment length polymorphism (AFLP) PCR test, digested samples were separated by native polyacrylamide gel electrophoresis with 10% (w/v) acrylamide using the Mini-PROTEAN Tetra Cell (BioRad). Samples containing equal volumes (10 μL) of PCR product were mixed with 6× Novel Juice loading dye (Sigma-Aldrich) and separated in a 1.0-mm-thick minigel (3.9 mL water, 0.6 mL 5× TBE, 1.5 mL 38:2 acrylamide:bis, 22.5 μL 10% [w/v] APS, 7.5 μL TEMED) in 0.5× TBE buffer at 50 V for 10 min and then increased to 100 V for 60 min (modified from [30]). Bands were visualized with the Azure c200 platform.

## Results

### Primer design, PCR, and multiplex

A total of 13 loci met the criteria established in our experimental design. In terms of potential gene candidates, the most stringent criterion was selecting single-copy orthologs. Applying all three criteria of our experimental design (single-copy genes, genetically diverse loci and *T*. *cruzi* species-specific loci) resulted in a total of 15 gene candidates, however two of these were not suitable for proper oligonucleotide design. This resulted in the selection of 15 MM (two loci had two distinct molecular tests designed for each loci). With regard to the function of the 13 candidate loci selected, some had a relationship to catalytic functions, such as TcSC5D, which is another *T*. *cruzi* MM that was developed in 2012 for the diagnosis of the disease [8]. In addition, there were loci involved in metabolic pathways for the biosynthesis of amino acids, membrane lipids and glycine, among other functions.

**Fig 1:**
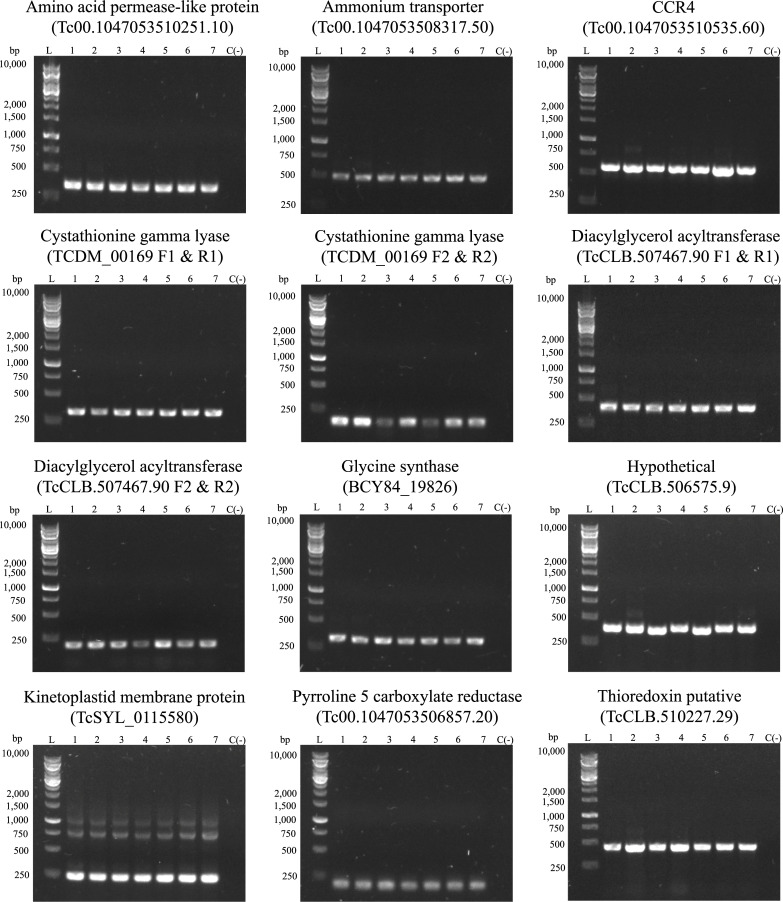
PCR analysis of MM selected. Lane number: (1) strain Tulacl2 (DTU VI); (2) strain PSC-O (DTU V); (3) strain EV13c (DTU I); (4) strain CANIII (DTU IV); (5) strain CA-1-05 (DTU I); (6) strain CBB cl3 (DTU II); (7) strain ESMclZ2 (DTU II). Negative control (C(-)). DNA ladder (L) (Fermentas, #SM1163).

The optimization of the primers designed resulted in only 12 MMs amplifying successfully, confirming their potential use in the molecular typing of the majority DTUs of the parasite (Table 1 and Fig 1).

**Table 1:** Characteristics of molecular markers selected.

Gene ID	Gene	Primer sequence (5’–3’)	PCR amplicon (nt)	Tm (°C)	SNPs
Tc00.1047053510251.10	Amino acid permease-like protein	F: TCTCTCTGGGACCATTCACG	350	58	15
		R: CGCCGTGAGACATGTAACAG			
Tc00.1047053508317.50	Ammonium transporter	F: TGCAGGTTTGGCTGGTATTAC	510	61	12
		R: TCTCCAATAAACAGGCTGCTG			
Tc00.1047053510535.60	ccr4-not transcription complex subunit	F: GAAGCCTGTGGGCAATTTTA	591	59	10
		R: GCGGAGCAGTTTGAAGTAGC			
TCDM_00169	Cystathionine gamma lyase	F1: GAAGCATTTGGTTTCCGACT	333	61	8
		R1: TGATCACCCGCACTCATAAA			
TCDM_00169	Cystathionine gamma lyase	F2: ATCACCGATTGCAGTTACGG	150	60	4
		R2: CGTCTCCGAAAGAACCAACT			
TcCLB.507467.90	Diacylglycerol acyltransferase	F1: TTGACAGTAGCACGCAGAGG	436	58	12
		R1: ACAGCACCTCGAGGGATTTA			
TcCLB.507467.90	Diacylglycerol acyltransferase	F2: CCCAACCGTGCCGTA	190	60	6
		R2: GGCCTTTGCGCGGTA			
BCY84_19826	Glycine synthase (GCVT1)	F: CGTTCTGCACAGTCTTCACC	331	59	6
		R: CCCCGCATTGATAACAGCAA			
TcCLB.506575.9	Hypothetical protein	F: GCGTACACAGGTAGCGAGTG	377*, 397^Φ^, 405 & 850^Ψ^, 383^Ω^	59	5
		R: AACCCACCAAAGAGGTGACA			
TcSYL_0115580	Kinetoplastid membrane protein	F1: ATGGCCACCACTCTTGAGG	238	61	6
		R1: ACTCAGCAAATTTGGCCTTG			
Tc00.1047053506857.20	Pyrroline 5-carboxylate reductase	F: ATGCCGTTTGTTCAGTGGTC	190	61	5
		R: GCGTTTGGTGTGACAGAAGT			
TcCLB.510227.29	Thioredoxin putative	F1: TTTTTGCGGAGGCACTTC	464	58	8
		R1: CGTGGCTTGGAAGAGAAGA			

Gene ID: nomenclature given to each gene as reported in TriTrypDB. Primer sequences are represented from 5’-3’. The PCR amplicon column shows the size predicted for each MM. Tm represents the ideal annealing temperature in centigrade degrees for each MM. SNPs represent the number of polymorphisms found within that MM by comparing the genetic sequence of the four distinct strains of *T*. *cruzi* used to select the MM

* predicted amplicon size for DTU I (monophyletic clade A)

^Φ^ predicted amplicon size for DTU II and IV (monophyletic clade C)

^Ψ^ predicted amplicon size for DTU V (monophyletic clades B and C)

^Ω^ predicted amplicon size for DTU VI (monophyletic clades B and C).

### Discrete typing unit identification

**Fig 2:**
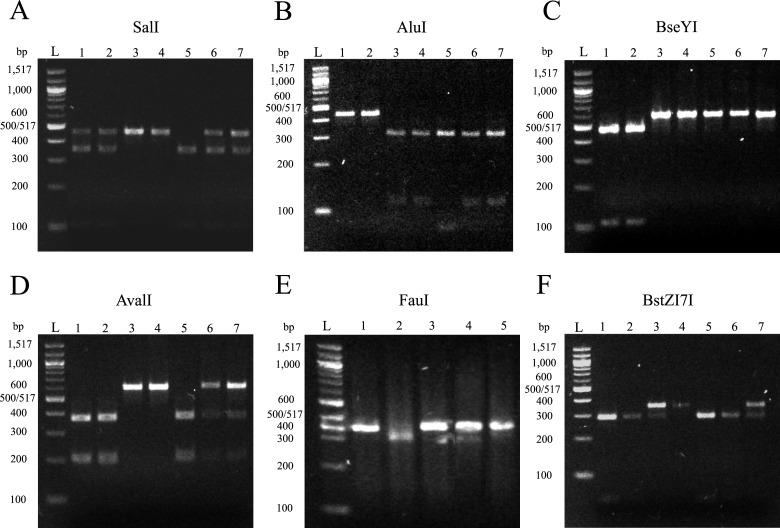
Electrophoresis pattern of the PCR-RFLP. Panel A: diacylglycerol acyltransferase (TcCLB.507467.90) digested with SalI. Panel B: diacylglycerol acyltransferase (TcCLB.507467.90) digested with AluI. Panel C: ccr4-not transcription complex subunit (Tc00.1047053510535.60) digested with BseYI. Panel D: ccr4-not transcription complex subunit (Tc00.1047053510535.60) digested with AvalI. Panel E: amino acid permease-like protein (Tc00.1047053510251.10) digested with FauI. Panel F: amino acid permease-like protein (Tc00.1047053510251.10) digested with BstZ17l. Lane numbers for panels A-D and F: (1) strain EV-13C (DTU I); (2) strain CA-1-05 (DTU I); (3) strain CBBcl3 (DTU II); (4) strain ESMcl3Z2 (DTU II); (5) strain CANIII (DTU IV); (6) strain PSC-O (DTU V); (7) strain Tulacl2 (DTU VI). Lane numbers for panel E: (1) strain Ev13C (DTU I); (2) strain ESMcl3Z2 (DTU II); (3) strain CANIII (DTU IV); (4) strain PSC-O (DTU V); (5) strain Tula cl2 (DTU VI). DNA ladder (L) (NEBioLabs, #N0551S).

The SNPs identified as potential restriction sites were used to select potential restriction enzymes that could differentiate among the major DTUs representing the known genetic diversity of the parasite. A total of eight restriction enzymes were found to be appropriate (S2 Table). Among the 12 effective MM, only the hypothetical protein (TcCLB.506575.9) had distinct gene sizes between the 3 DTUs compared. Two MMs had restriction sites appropriate for the differentiation of four DTUs of the parasite (ccr4-not transcription complex subunit (Tc00.1047053510535.60) and diacylglycerol acyltransferase (TcCLB.507467.90)), while one MM could only discriminate DTU II (amino acid permease-like protein (Tc00.1047053510251.10)) (Table 2). The theoretical digested fragment sizes were previsualized in Serial Cloner 2.6.1. and were corroborated in the lab (Fig 2) using DNA from *T*. *cruzi* clones kindly provided by Dr. Carlos Machado at the University of Maryland (S1 Table).

**Table 2:** Digestion fragment patterns for the identification of DTUs.

Molecular marker	DTU (monophyletic clade)					Restriction enzyme
	DTU I (A)	DTU II (C)	DTU IV(D)	DTU V (B & C)	DTU VI (B & C)	
ccr 4 (Tc00.1047053510535.60)	478 & 113	No cut	No cut	No cut	No cut	BseYI
	373 & 218	No cut	373 & 218	373 & 218	373 & 218	AvalI
diacylglycerol acyltransferase (TcCLB.507467.90)	338 & 98	No cut	338 & 98	338 & 98	338 & 98	SalI
	No cut	314 &122	314 &122	314 &122	314 &122	AluI
amino acid permease-like protein (Tc00.1047053510251.10)	289 & 61	No cut	289 & 61	289 & 61	289 & 61	BstZ17I*
	No cut	293 & 57	No cut	No cut	No cut	FauI

Numbers in cells represent the digestion fragment sizes in base pairs of DNA. “No cut” means the enzyme should not cause a digestion of the original PCR amplicon; therefore, the original PCR amplicon size (see Table 1) should be observed. *The enzyme AccI can be used in replacement of BstZ17I; however, AccI is more ambiguous with respect to the target site. Thus, the use of BstZ17I is recommended. TcIII is not included since no genome or DNA was available in our lab at the time of the study. Monophyletic clade A represents DTU I strains, B represents strains classified as DTU II, V & VI (since DTU V & VI are hybrids of DTUII and DTUIII ancestors), C represents strains of DTU III, V & VI, and monophyletic clade D represents strains classified as DTU IV.

The digestion profiles confirmed the theoretical digestions (Fig 2 and Table 2).

Both the ccr4-not transcription complex subunit (Tc00.1047053510535.60) and the F1/R1 diacylglycerol acyltransferase (TcCLB.507467.90) MM are able to independently differentiate DTU I, DTU II, DTU IV, and DTU V/VI from each other (Table 2, Fig 2A–2D).

**Fig 3:**
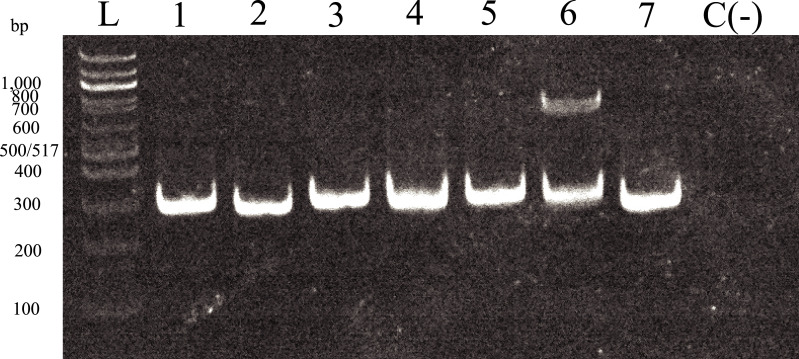
AFLP-PCR of TcCLB.506575.9 MM. Lane number: (1) strain EV-13C (DTU I), 377 bp amplicon; (2) strain CA-1-05 (DTU I), 377 bp amplicon; (3) strain CBBcl3 (DTU II), 397 bp amplicon; (4) strain ESMcl3Z2 (DTU II), 397 bp amplicon; (5) strain CANIII (DTU IV), 397 bp amplicon; (6) strain PSC-O (DTU V), 405 & 850 bp amplicons; (7) strain Tulacl2 (DTU VI), 383 bp amplicon. DNA ladder (L) (NEBioLabs, #N0551S).

The amplified fragment length polymorphism (AFLP-PCR) of the hypothetical protein (TcCLB.506575.9) yields different PCR amplicon sizes for at least four distinct DTUs. This amplified a 377 bp fragment for DTU I, a 397 bp segment for DTUs II and IV, a 405 bp fragment for DTU V, and a 383 bp segment for DTU VI, with an additional nonspecific amplicon of approximately 850 bp for DTU V. These results corroborated the capacity of this particular MM in discriminating four of the six DTUs of *T*. *cruzi* without the need to digest the amplicon (Fig 3).

### Effectivity in sylvatic samples

To compare the effectiveness of the proposed MM with the previously published ones, a frequently used and cited PCR-based diagnosis was included (TcSC5D gene) [8, 31]. The test was conducted using DNA extracted from *T*. *cruzi* clones and field-collected infected bugs (*Dipetalogaster maxima*). All results (both TcSC5D MM and the newly proposed MM) were positive, which highlighted the effectiveness of these MMs (S1 and S2 Figs).

The results are indicative of a lower PCR yield from DNA extracted from sylvatic specimens versus DNA clones (S1 Fig). This pattern was consistent with the previously published MM [8]. Comparing the PCR yield of a commonly used MM [8] and the MMs selected in this study, these yields appear comparable, with the exception of a few of the proposed MMs that appear to have higher yields (ammonium transporter (Tc00.1047053508317.50), both diacylglycerol acyltransferase markers (TcCLB.507467.90), the ccr4-not transcription complex subunit (Tc00.1047053510535.60), and the hypothetical protein used as the AFLP-PCR marker (TcCLB.506575.9)) (S1 Fig).

## Discussion

### Genome approach

A complete picture of the genetic background of *T*. *cruzi* has been partially explored [11, 12], since currently the genomes of a limited number of strains of the parasite have been sequenced and novel genetic diversity from nature continues to be described [11, 32]. A complete representation of the genomic sequences of all DTUs is still lacking, although the genomic data that are currently available for *T*. *cruzi* are not insignificant [15–17]. Thus, the annotated genomic sequences of four distinct strains of *T*. *cruzi* were used to assist selection of MM with the traits needed to differentiate the DTUs of the parasite. Moreover, multiple thorough phylogenetic analyses condense the current six-DTU classification system employed for *T*. *cruzi* into a classification of three to four monophyletic clades [13, 14]. This implies that the DTUs represented in this study cover three of the four monophyletic clades that have thus far been described for this parasite in nature.

Therefore, the initial goal in this study was to find multiple MM that would differ in the PCR amplicon sizes among major DTUs (AFLP-PCR), which would avoid the use of the enzymatic digestion of the PCR amplicon, making the diagnosis faster, easier, and cheaper. This objective was achieved with only the hypothetical protein marker (TcCLB.506575.9). Two additional MMs were selected with the ability to differentiate DTUs I, II, IV and V/VI with an additional RFLP approach.

Applying the most appropriate criteria of traits for our goal led to the selection of the potential loci. The criterion of finding single-copy orthologs was the most stringent. After all our criteria were applied, 13 loci were selected. The protein functions of the selected loci varied. The functions included catalytic enzymes, intracellular transport of amino acids, cell membrane structure, intracellular vesicles, oxidoreductase, ammonium transporter, and a gene with unknown function (Table 1).

### PCR-RFLP and AFLP-PCR validation

Of the 15 MMs selected, 12 were successfully amplified in the PCR reaction (Fig 1). Three MMs could not be amplified, which could be due to the following reasons: (A) the presence of SNPs in the annealing regions in the DNA strains used, since not all polymorphisms could be detected in silico, given the actual genetic diversity available, (B) undetected primer dimer formation of the oligonucleotides, and (C) annealing temperatures much lower than those used in the initial primer characterization. In terms of the specificity of the primers, a specificity test (S2 Fig) confirmed the singularity of the MM, specific to the *T*. *cruzi* genome. Furthermore, a bioinformatics analysis confirmed the presence of multiple SNPs in the annealing primer regions of closely related species of the genus *Trypanosoma* (Table 3).

**Table 3:** Species specificity of primers within *Trypanosoma* genus.

	ccr 4 (Tc00.1047053510535.60)	diacylglycerol acyltransferase (TcCLB.507467.90)	amino acid permease-like protein (Tc00.1047053510251.10)	Hypothetical protein (TcCLB.506575.9)
***T*. *cruzi***	0/0	0/0	0/0	0/0
***T*. *rangeli***	11/11.	NA	5/8.	2/3
***T*. *vivax***	4/3.	NA	14/9.	5/5
***T*. *evansi***	NA/6	8/NA	12/9.	NA
***T*. *theileri***	5/2.	10/6.	7/9.	4/5
***T*. *marinkellei***	4/5.	2/2.	1/3.	1/3

Columns represent the number of SNPs present in the annealing zones for the forward and reverse primers, respectively, in the ccr4-not transcription complex subunit (Tc00.1047053510535.60), diacylglycerol acyltransferase (TcCLB.507467.90), amino acid permease-like protein (Tc00.1047053510251.10), and the hypothetical-like protein (TcCLB.506575.9) MMs.

NA: annealing zone could not be located in the species. Since either the absence of the homologous region or the homologous region is so divergent, it can no longer be identified by BLAST.

Of the 12 MMs that were successful in their respective PCR amplifications, two had the genomic diversity needed to select restriction enzymes that would produce different digestion patterns for the distinction of four DTUs (I, II, IV and V/VI) out of the six current DTUs (Fig 2A–2D), while 1 MM could only differentiate between two DTUs (Fig 2E and 2F). All MM confirmed the in silico digestion fragment sizes predicted. Furthermore, the efficiency of the PCR amplification was compared to a recent and commonly used PCR MM (S1 Fig) [8]. The results clearly showed that the markers in this study provide an additional typing test for *T*. *cruzi*. One of the MMs selected in this study can differentiate, with AFLP-PCR, among four distinct DTUs (Fig 3). The electrophoresis analysis of this MM (hypothetical protein: TcCLB.506575.9) revealed that the PCR is not 100% specific in DTU V, since an additional weak amplicon was observed, most likely due to the presence of a paralogous copy of this gene. This has the potential to increase the diagnostic ability of this particular MM. As a result, we recommend using either the ccr4-not transcription complex subunit (Tc00.1047053510535.60) MM with BseYI and AvaII or the diacylglycerol acyltransferase (TcCLB.507467.90) MM with SalI and AluI (Table 2), since these two markers appear to have the highest resolution of all twelve selected MM.

It should not be surprising that the PCR yield is much lower when the template DNA is extracted from sylvatic specimens, compared to in vitro clones. The lower presence of parasites in sylvatic infections will result in a reduced quantity of template DNA for the PCR. This is especially clear in S2 Fig. In every case (including the control TcSC5D MM and our MMs), the PCR bands that were derived from *T*. *cruzi* clone DNA were much stronger than the amplifications using sylvatic DNA templates. This is one of the reasons why many studies usually target a genomic region that might have multiple copies per cell (e.g., kinetoplast chromosomes) [33, 34]. In this study a simple solution could have been to select a paralog gene instead of orthologs. This would have been a suitable approach if the main goal were to simply diagnose the infection, since the larger amount of genetic copies found within the genome would translate into a higher yield from PCR. However, finding a paralog that would produce a digestion pattern unique for each DTU is very unlikely, given the large number of repetitive regions characteristic of *T*. *cruzi* [15].

This study focused on selecting single-copy orthologs as targets. This strategy would ultimately allow the identification of the genetic background of the parasite, which was the ultimate objective of the study. A setback to the study was the lower PCR yield compared to targeting a multiple-copy DNA region.

### The ability to identify DTUs of *T*. *cruzi*

Since the genetic diversity of *T*. *cruzi* is currently categorized in six DTUs and recent studies suggest that there might be an even larger amount of genetic diversity [12], resolution of the MM in discriminating all DTUs cannot currently be achieved. When a more complete understanding of the real genetic diversity of the parasite has been described, a broader genomic search for better MM will be possible. However, many studies have consistently shown that the genetic diversity of the parasite is not necessarily naturally divided into seven DTUs [13, 14, 35, 36]; it more closely assembles into three or four genetic clusters, depending on whether a mitochondrial or a nuclear marker is used. Further application of this division would reclassify the genetic diversity of the parasite into four or five DTUs (since TcBat has usually not been included in the three or four genetic clade studies), and this in turn would mean these markers could still be valid and able to distinguish types within the main genetic diversity of the parasite.

## Conclusions

Genomic sequences were successfully used to design molecular tests with an ability to identify the genetic background of *T*. *cruzi* without the need to perform any sequencing reaction. Although the complete genetic diversity of this neglected disease has not been completely explored, the available genomic sequence was vital for the development of novel molecular tests and their potential application for diagnostic purposes. When a more comprehensive description of all the genetic diversity of *T*. *cruzi* is accessible, the development of additional and more efficient genetic markers will be possible.

Future work should focus on the application of the MM described in clinical samples. Part of this work is currently being done in collaboration with Centro Regional de Investigación en Salud Pública, Chiapas, México.

## Supporting information

S1 Fig PCR yield in biological versus clone-derived DNA. Numbers with apostrophe (e.g., 2’) represent PCR amplicons derived from cultured T. cruzi DNA (DTU II strain CBBcl3), whereas numbers without apostrophe represent PCR amplicons derived from DNA extracted from field-collected specimens of Dipetalogaster maxima, previously diagnosed in our lab as positive for T. cruzi. L: DNA ladder (BioTang, UMR-150). Lanes: (1) TcSC5D; (2) amino acid permease-like protein (Tc00.1047053510251.10); (3) ammonium transporter (Tc00.1047053508317.50); (4) CCR 4 (Tc00.1047053510535.60); (5) cystathionine gamma lyase 1 (TCDM_00169 F1 & R1); (6) cystathionine gamma lyase (TCDM_00169 F2&R2); (7) diacylglycerol acyltransferase 1 (TcCLB.507467.90 F1 & R1); (8) diacylglycerol acyltransferase 2 (TcCLB.507467.90 F2 & R2); (9) glycine synthase (BCY84_19826); (10) hypothetical protein (TcCLB.506575.9); (11) kinetoplastid membrane protein (TcSYL_0115580); (12) pyrroline 5 carboxylate reductase (Tc00.1047053506857.20); (13) thioredoxin putative (TcCLB.510227.29).
(EPS)

S2 Fig Specificity of primers on non-*T*. *cruzi* DNA. Numbers from one to six represent PCR amplicons derived from DNA extracted from field-collected specimens. The seventh position indicates PCR amplicon derived from culture-derived *T*. *cruzi* DNA (DTUII strain CBBcl3). C (-) stands as negative control. L1: DNA ladder (Fermentas, #SM1163).
(EPS)

S1 Table Information of culture-derived DNA strains. *T*. *cruzi* DNA strains used in this study. The geographical and host origin of the sample are reported, as well as its monophyletic classification based on previous studies [13, 14]. All the DNA samples were kindly donated by Dr. Carlos Machado.
(DOCX)

S2 Table Characteristics of the selected restriction enzymes. Name of the restriction enzyme, target sequence, cut position, and recommended buffer for optimal activity.
(DOCX)

S1 Raw images (PDF)
