# Supplementary material for: Design of a AFLP-PCR and PCR-RFLP test that identify the majority of discrete typing units of Trypanosoma cruzi
Source: PLoS One. 2020 Aug 4;15(8):e0237180. doi: 10.1371/journal.pone.0237180 (PMC7402520; doi:10.1371/journal.pone.0237180)
Supplement: S1 Table — T. cruzi DNA strains used in this study. The geographical and host origin of the sample are reported, as well as its monophyletic classification based on previous studies [13, 14]. All the DNA samples were kindly donated by Dr. Carlos Machado. (DOCX) [file pone.0237180.s003.docx]

**Supplementary Table 1. Information of culture-derived DNA strains**

| **Strain** | **DTU** | **Source** | **Monophyletic clade** |
| --- | --- | --- | --- |
| CA-1-05 | TcI | (Brazil, *Triatoma pseudomaculata*) | A |
| EV-13C | TcI | (Colombia, *Rhodnius prolixus*) | A |
| CBBcl3 | TcII | (Chile, human) | C |
| ESMcl3Z2 | TcII | (Brazil, human) | C |
| CANIII | TcIV | (Brazil, human) | D |
| PSC-O | TcV | (Chile, human) | B & C |
| Tulacl2 | TcVI | (Chile, human) | B & C |
