# Supplementary material for: Design of a AFLP-PCR and PCR-RFLP test that identify the majority of discrete typing units of Trypanosoma cruzi
Source: PLoS One. 2020 Aug 4;15(8):e0237180. doi: 10.1371/journal.pone.0237180 (PMC7402520; doi:10.1371/journal.pone.0237180)
Supplement: S2 Table — Name of the restriction enzyme, target sequence, cut position, and recommended buffer for optimal activity. (DOCX) [file pone.0237180.s004.docx]

**Supplementary Table 2.** **Characteristics of the selected restriction enzymes**

| **CCR 4** | **Sequence 5' a 3'** | **Sequence 3' a 5'** | **Position** | **Buffer** |
| --- | --- | --- | --- | --- |
| BseYI | CCCAGC | GGGTCG | 478 | 3 |
| AvaII | GGWCC | CCWGG | 373 | Cut smart |
|  |  |  |  |  |
| **Diacylglycerol acyltransferase** |  |  |  |  |
| AluI | AGCT | TCGA | 314 | Cut smart |
| SalI | GTCGAC | CAGCTG | 338 | 3 |
| Sau96I | GGNCC | CCNGG | 336 | Cut smart |
|  |  |  |  |  |
| **Amino acid permease-like protein** |  |  |  |  |
| BstZ17I | GTATAC | CATATG | 289 | Cut smart |
| AccI | GTMKAC | CAKMTG | 289 | Cut smart |
| FauI | CCCGC | GGGCG | 293 | Cut smart |
