# Supplementary material for: Design of a AFLP-PCR and PCR-RFLP test that identify the majority of discrete typing units of Trypanosoma cruzi
Source: PLoS One. 2020 Aug 4;15(8):e0237180. doi: 10.1371/journal.pone.0237180 (PMC7402520; doi:10.1371/journal.pone.0237180)
Supplement: S1 Raw images — (PDF) [file pone.0237180.s005.pdf]

Fig 1. PCR analysis of MM selected.

Note: The following lane order is the same for all the figures included in this bullet (Fig 1). The images were all visualized and captured with Azure c200 platform.

Lane order: (1) strain Tulacl2 (DTU VI); (2) strain PSC-O (DTU V); (3) strain EV13c (DTU I); (4) strain CANIII (DTU IV); (5) strain CA-1-05 (DTU I); (6) strain CBB cl3 (DTU II); (7) strain ESMclZ2 (DTU II). Negative control). DNA ladder (L) (Fermentas, #SM1163).

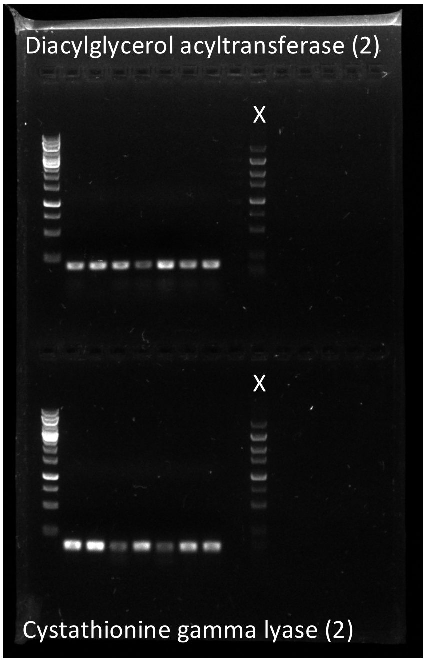

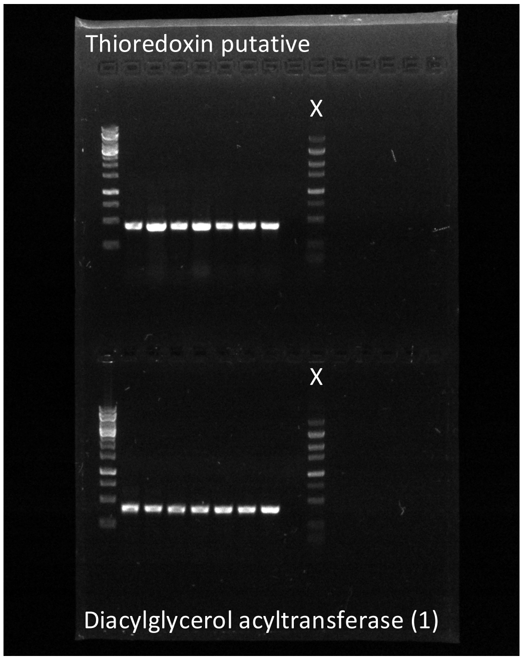

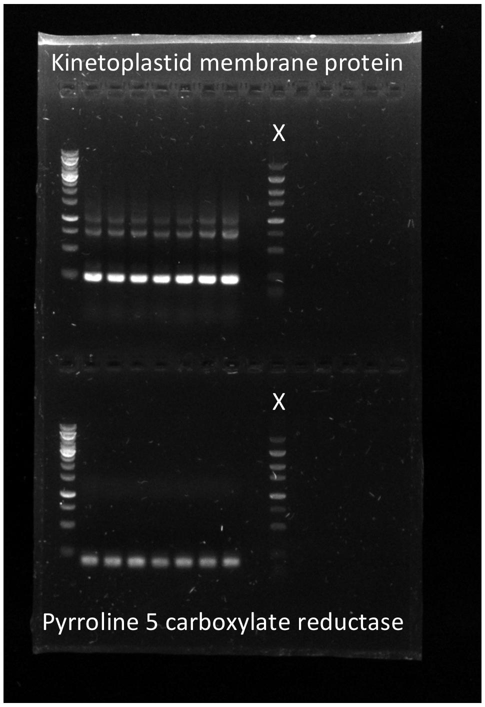
Kinetoplastid membrane protein
X
X
Pyrroline 5 carboxylate reductase

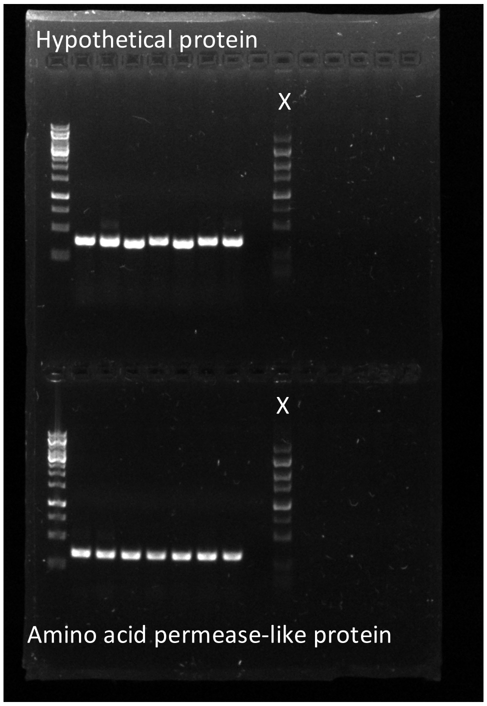
Hypothetical protein
X
X
Amino acid permease-like protein

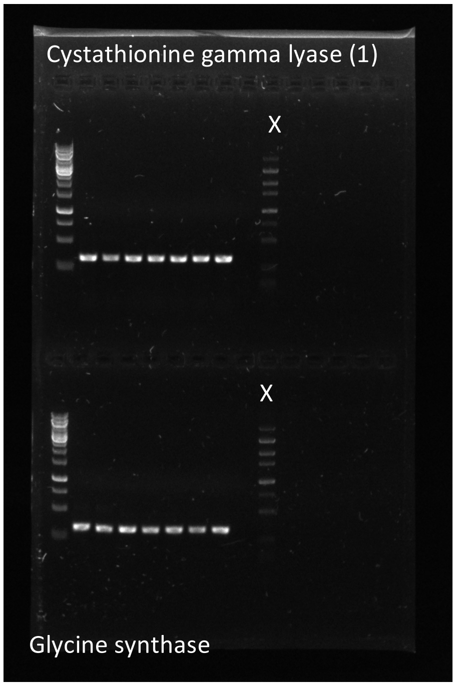
Cystathionine gamma lyase (1)
X
X
Glycine synthase

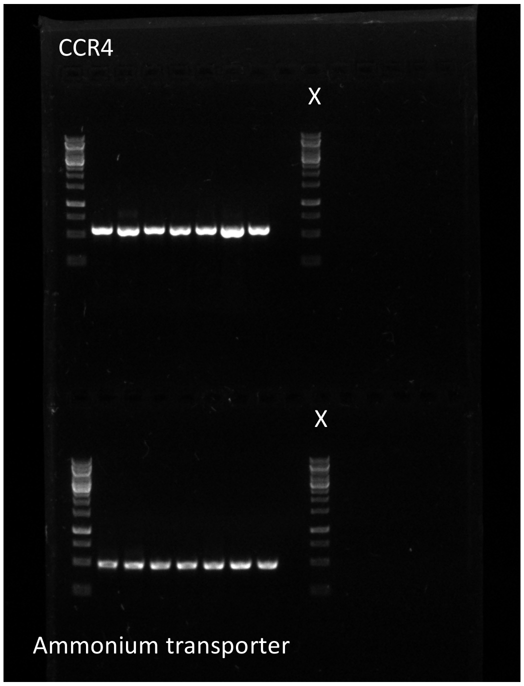
CCR4
X
X
Ammonium transporter

Figure 2. Electrophoresis pattern of the PCR-RFLP

The images were all visualized, capture and edited with Azure c200 platform.

Lanes orders for panel A-D and F: (1) strain EV-13C (DTU I); (2) strain CA-1-05 (DTU I); (3) strain CBBcl3 (DTU II); (4) strain ESMcl3Z2 (DTU II); (5) strain CANIII (DTU IV); (6) strain PSC-O (DTU V); (7) strain Tulacl2 (DTU VI).
Lane order for panel E: (1) strain Ev13C (DTU I); (2) strain ESMcl3Z2 (DTU II); (3) strain CANIII (DTU IV); (4) strain PSC-O (DTU V); (5) strain Tula cl2 (DTU VI). DNA ladder (L) (New England BioLabs, N0551S).

Panel A, lane 1-5: Diacylglycerol acyltransferase (TcCLB.507467.90) digested with SalI.
Panel B, lane 6-10: Diacylglycerol acyltransferase (TcCLB.507467.90) digested with AluI.

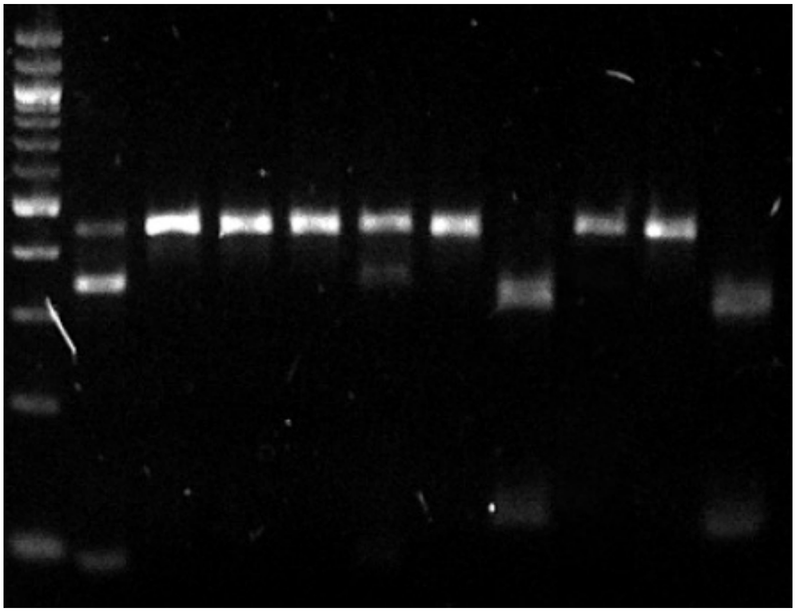

Panel C, lane 1-5: CCR4-nottranscription complex subunit (Tc00.1047053510535.60) digested with BseYI.
Panel D, lane 6-10: CCR4-not transcription complex subunit (Tc00.1047053510535.60) digested with AvaII.

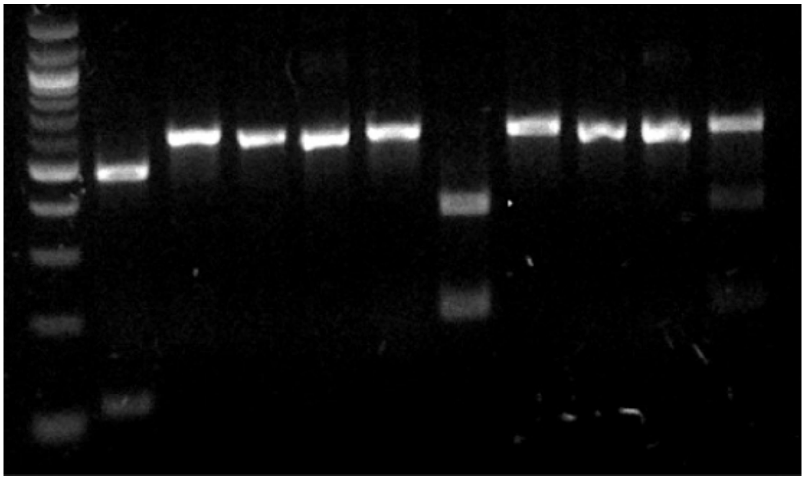

Panel E, lane 1-5: Amino acid permease-like protein (Tc00.1047053510251.10) digested with FauI.
Panel F, lane 6-10: Amino acid permease-like protein (Tc00.1047053510251.10) digested with BstZ17I.

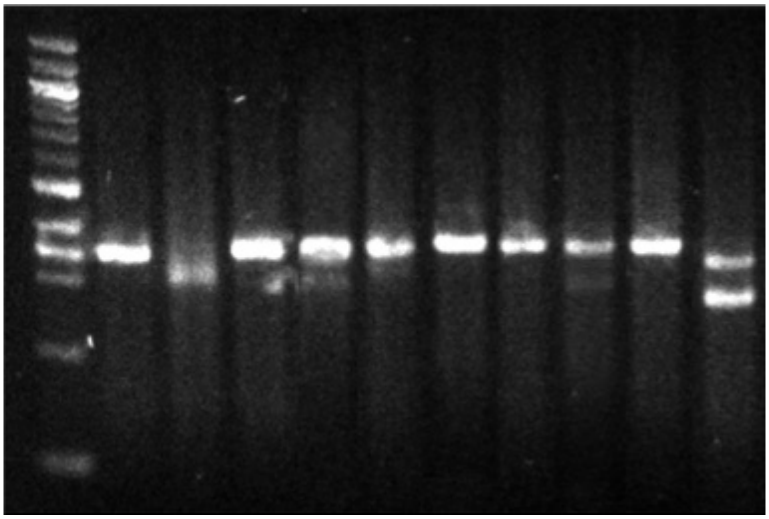

Figure 3. AFLP-PCR of TcCLB.506575.9 MM

The image was visualized and capture with Azure c200 platform.

Lane order: (1) strain EV-13C (DTU I), 377 bp amplicon; (2) strain CA-1-05 (DTUI), 377 bp amplicon; (3) strain CBBcl3 (DTU II), 397 bp amplicon; (4) strain ESMcl3Z2 (DTU II), 397 bp amplicon; (5) strain CANIII (DTU IV), 397 bp amplicon; (6) strain PSC-O (DTU V), 405 & 850 bp amplicons; (7) strain Tulacl2 (DTU VI), 383 bp amplicon. DNA ladder (L) (NEBioLabs, #N0551S).

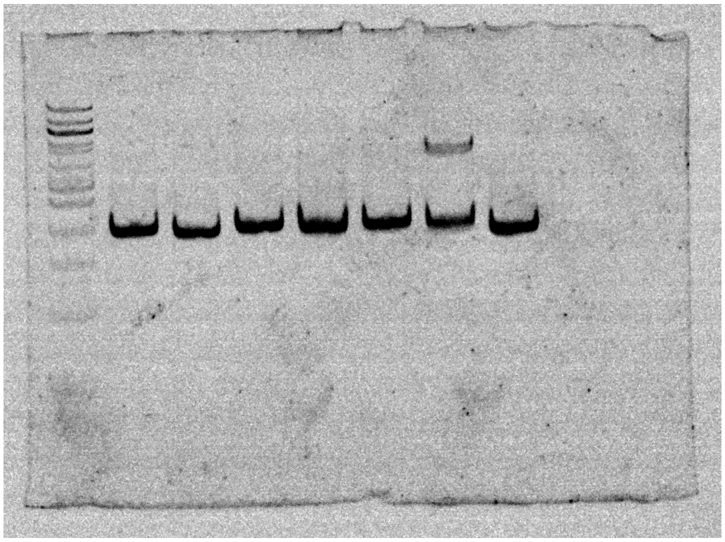

Supporting Information

Supplementary Figure 1. PCR yield in biological versus clone-derived DNA

The images were visualized, capture and edited with Azure c200 platform.

Note: Numbers with apostrophe (e.g., 2') represent PCR amplicons derived from cultured *T. cruzi* DNA (DTU II strain CBBcl3), whereas numbers without apostrophe represent PCR amplicons derived from DNA extracted from field-collected specimens positive for *T. cruzi;* except for the lanes 1 and 1', which are inverse.

Lane order: (1) TcSC5D; (2) amino acid permease-like protein (Tc00.1047053510251.10); (3) ammonium transporter (Tc00.1047053508317.50); (4) CCR 4 (Tc00.1047053510535.60); (5) cystathionine gamma lyase 1 (TCDM_00169 F1 & R1); (6) cystathionine gamma lyase (TCDM_00169 F2 & R2); (7) diacylglycerol acyltransferase 1 (TcCLB.507467.90 F1 & R1); (8) diacylglycerol acyltransferase 2 (TcCLB.507467.90 F2 & R2); (9) glycine synthase (BCY84_19826); (10) hypothetical protein (TcCLB.506575.9); (11) kinetoplastid membrane

protein (TcSYL_0115580); (12) pyrroline 5 carboxylate reductase (Tc00.1047053506857.20); (13) thioredoxin putative (TcCLB.510227.29). DNA ladder (BioTang, UMR-150).

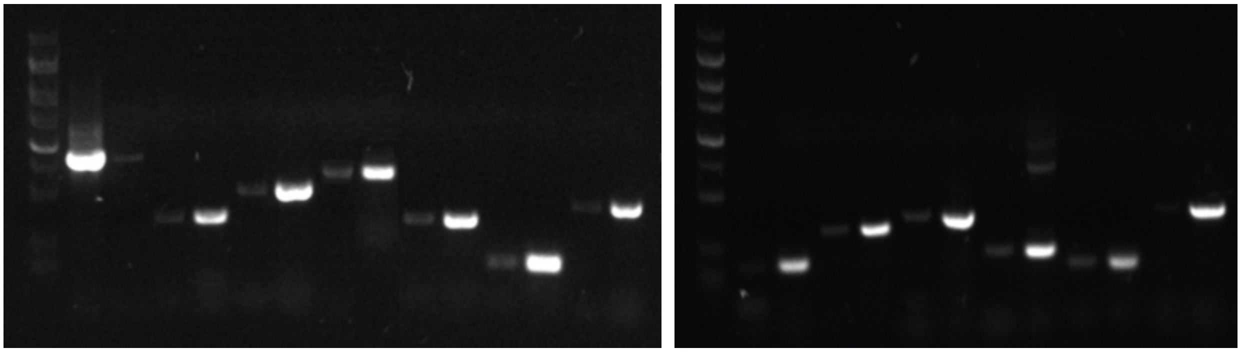

Supplementary Figure 2. Specificity of primers on non-T. cruzi DNA

The images were all visualized and capture with Azure c200 platform.

Lanes from one to six represent PCR amplicons derived from DNA extracted from field-collected specimens. The last position indicates PCR amplicon derived from culture-derived *T. cruzi* DNA (DTUII strain CBBcl3). L1: DNA ladder (Fermentas, #SM1163).

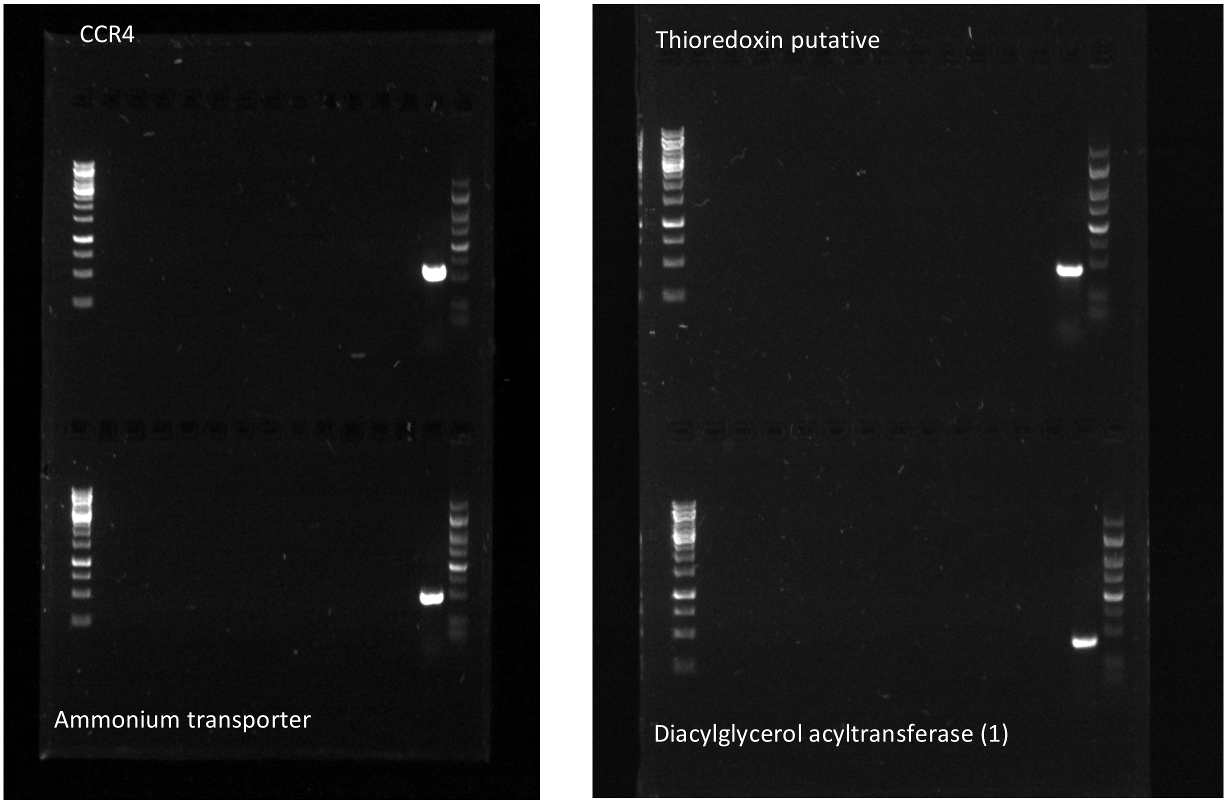

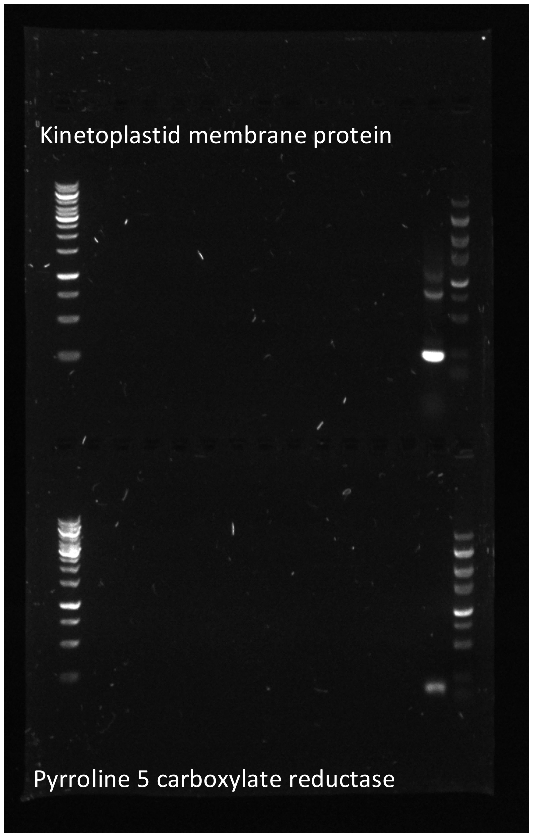
Kinetoplastid membrane protein
Pyrroline 5 carboxylate reductase

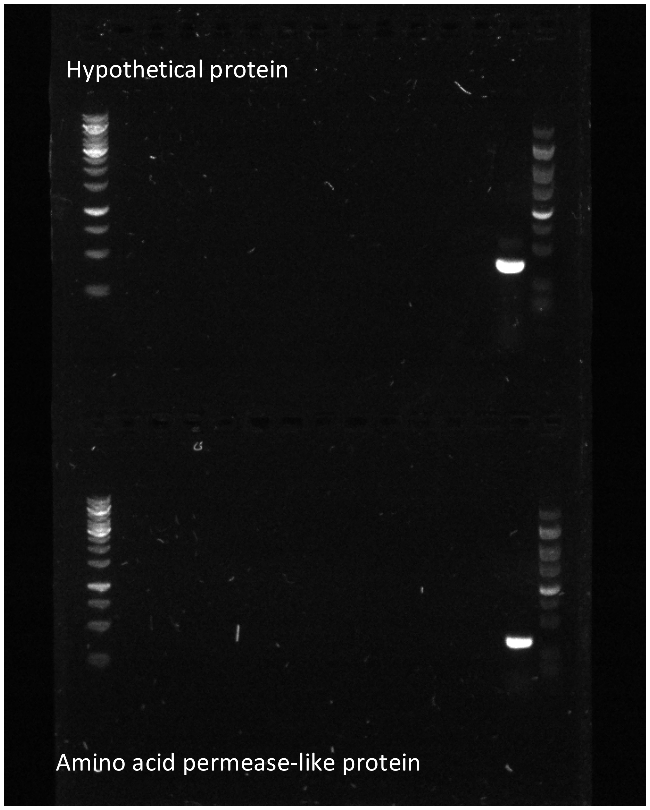
Hypothetical protein
Amino acid permease-like protein

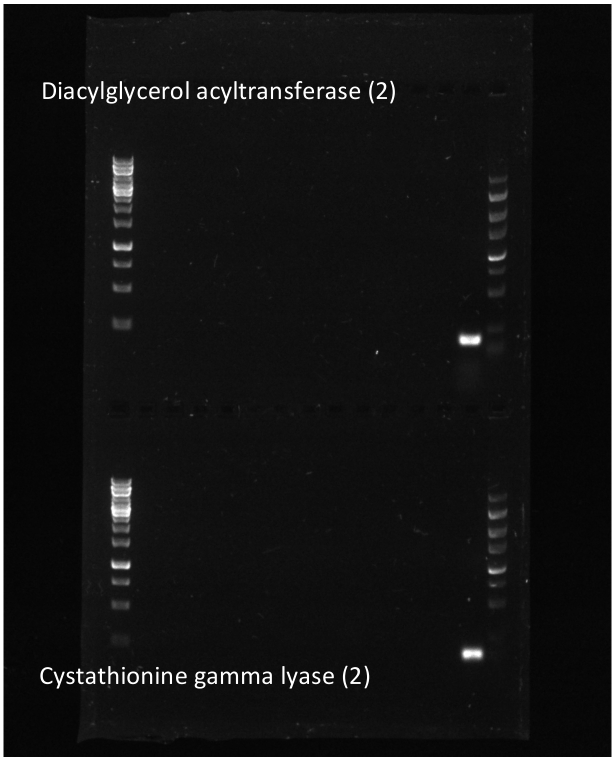
Diacylglycerol acyltransferase (2)
Cystathionine gamma lyase (2)

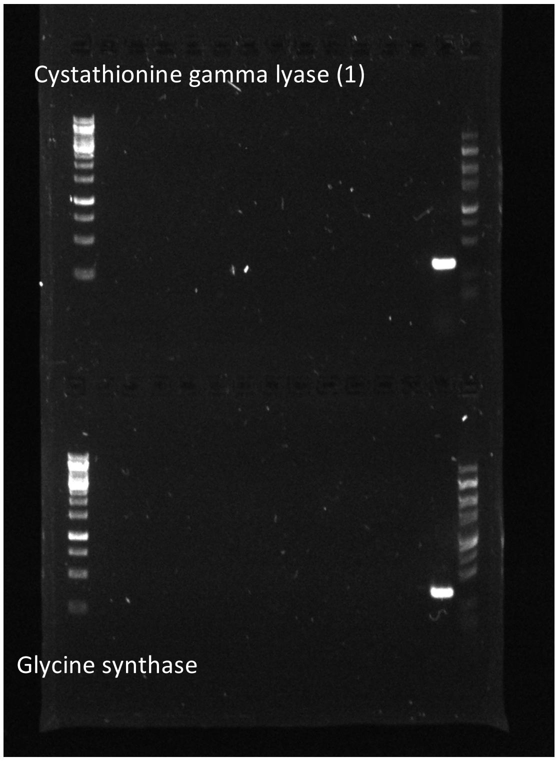
Cystathionine gamma lyase (1)
Glycine synthase
